# Elements of fish metacommunity structure in Neotropical freshwater streams

**DOI:** 10.1002/ece3.6804

**Published:** 2020-10-15

**Authors:** Thiago Bernardi Vieira, Leandro Schlemmer Brasil, Liriann Chrisley N. da Silva, Francisco Leonardo Tejerina‐Garro, Pedro de Podestà Uchôa de Aquino, Paulo S. Pompeu, Paulo de Marco

**Affiliations:** ^1^ Laboratório de Ictiologia de Altamira Faculdade de Ciências Biológicas Universidade Federal do Pará Altamira Pará Brazil; ^2^ Programa de Pós‐graduação em Zoologia Universidade Federal do Pará Belém Pará Brazil; ^3^ Centro de Biologia Aquática Escola de Ciências Agrárias e Biológicas Pontifícia Universidade Católica de Goiás Goiânia Goiás Brazil; ^4^ Programa de Pós‐Graduação em Sociedade Tecnologia e Meio ambiente UniEVANGÉLICA Anápolis Goiás Brazil; ^5^ Departamento de Zoologia Universidade de Brasília Brasília Distrito Federal Brazil; ^6^ Departamento de Biologia Universidade Federal de Lavras Lavras Minas Gerais Brazil; ^7^ Departamento de Ecologia Instituto de Ciências Biológicas Universidade Federal de Goiás Campus II Goiânia Goiás Brazil

## Abstract

The identification of the mechanisms underlying patterns of species co‐occurrence is a way to identify which process(es) (niche, neutral, or both) structure metacommunities. The current paper had the goal of identifying patterns of co‐occurrence in Neotropical stream fish and determining which processes structure the fish metacommunity, and identifying any gradients underlying this structure. Results indicated that the metacommunity formed by the species pool was structured by a pattern of nested co‐occurrence (hyperdispersed species loss) and a mass‐effect mechanism. However, a set of core species, displaying a Clementsian pattern, was structured by a species‐sorting mechanism. Both, hyperdispersed species loss and the Clementsian patterns point to a discrete set of communities within the metacommunity. These communities could be isolated by the water physicochemical conditions or morphological characteristics of the stream channel.

## Graphical abstract

The results of the work show a set of main species of stream fish with a Clementian pattern and structured by a *species* classification mechanism. On the other hand, the metacommunity formed by the total set of species is structured by a nested pattern (loss of hyperdispersed species) and by the mass‐effect mechanism. These communities can be isolated by physical–chemical conditions or physical barriers.

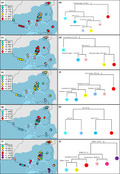

## 1 INTRODUCTION

Patterns of species co‐occurrence in metacommunities result from interactions between them, environment, and/or neutral dynamics (Brasil et al., 2017; Fernandes et al., 2013; Leibold et al., 2004; Leibold & Mikkelson, 2002; López‐González et al., 2012; Presley et al., 2012). Species co‐occurrence is a response to environmental conditions (e.g., morphological structure, physical, chemical, physicochemical, and climatic conditions) and resources (e.g., food, predation, and reproduction) of the habitat, mediated by the dispersal abilities of the species pool (Leibold et al., 2004; Leibold & Mikkelson, 2002; Presley & Willig, 2010; Urbieta et al., 2014). Between‐species differences in dispersion capacity imply the existence of filters and/or barriers in the geographic space, posing questions relating to matrix (background ecological system) permeability (Fernandes et al., 2013; Kennedy et al., 2014).

Interaction between local (habitat physical conditions and resources) and regional factors (matrix permeability) in the metacommunity involves four main mechanisms: (a) patch dynamics (PD); (b) species sorting (SS); (c) mass effects (ME), and (d) neutrality (NE: Leibold et al., 2004). Use of this framework has allowed multidisciplinary analysis of metacommunity dynamics across landscapes, combining theoretical concepts established over several decades (Falke & Fausch, 2010; Leibold et al., 2004; Leibold & Mikkelson, 2002; Peres‐Neto & Cumming, 2010; Presley & Willig, 2010), such as competition (Connor & Simberloff, 1979; Simberloff, 1983; Tilman, 1982), species nesting (Patterson, 1986), and species distribution patterns (Clements, 1916; Gleason, 1926). This has allowed the metacommunity concept to become a framework for studies in ecology aiming to understand species co‐occurrence patterns.

In the PD mechanism, the environment is viewed as a mosaic of habitat patches, all of which are equal in quality and availability; environmental filters are considered nonoperational, with all species of the regional pool having a high potential for dispersion and competition (Leibold et al., 2004). The PD can be presented as a checkerboard pattern, where between‐species interactions (competition and facilitation) are the only operational explanation. The NE mechanism suggests that the metacommunity has a pool of ecologically identical species and co‐occurrence is a product of stochastic processes along with colonization and extinction (Leibold et al., 2004). A random pattern of co‐occurrence is predicted when an NE mechanism is operating (Astorga et al., 2011; Ulrich & Zalewski, 2006; Vinson & Hawkins, 2003). Identification of PD and NE as metacommunity structuring mechanisms occurs by assessing the relationships between the species pool and the regional environmental conditions, usually represented by the geographical distance between communities (Cottenie, 2005).

In contrast, SS and ME are mechanisms related to the niche dynamics (Göthe et al., 2013), represented by an environmental gradient and the species–environment relationships, with differences occurring in the dispersal abilities of the species pool (Leibold et al., 2004). SS mechanisms consider species as weak dispersers (species disperse within the metacommunity, but the dispersion cannot modify the observed co‐occurrence pattern), so that a species distribution within the metacommunity is only related to local conditions (Leibold et al., 2004). ME mechanisms operate when species present in the pool have high dispersal abilities, so increasing the colonization, decreasing local extinction rates, and modifying the co‐occurrence pattern (Leibold et al., 2004). As a result, ME is characterized by interactions between local and regional factors structuring the species pool distribution and a nested species distribution within the metacommunity.

**Figure 1:**
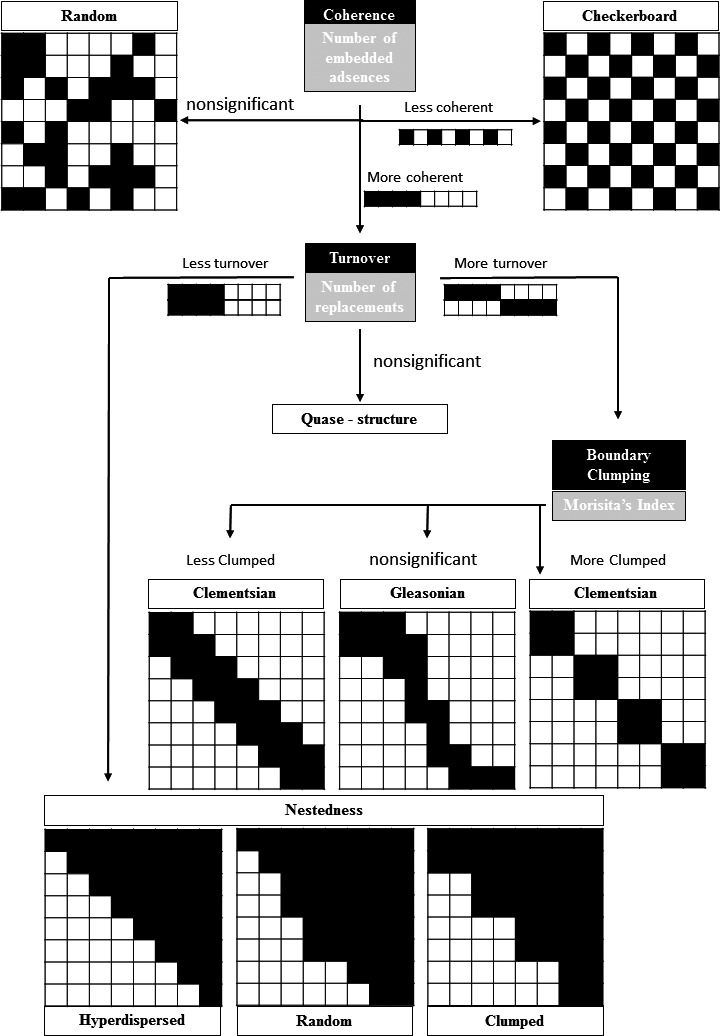
Relationship between the Elements of Metacommunity Structure (EMS) and within‐metacommunity species co‐occurrence patterns. Adapted from Leibold and Mikkelson (2002) and Presley et al. (2010)

Metacommunity species distribution patterns can be identified by the presence of three elements: (a) coherence, (b) turnover, and (c) boundary clumping, which together comprise the metacommunity structure (EMS), and can be identified by the double ordination of the species’ incidence matrix (coherence and turnover) and Morisita's overlap index (boundary clumping; Leibold & Mikkelson, 2002). Coherence is assessed by the number of absences incorporated in the matrix; a greater number of embedded absences is indicative of low coherence in the matrix (Figure 1; Leibold & Mikkelson, 2002). Turnover is measured by the substitution of one species by another at a pair of sites (Figure 1), with boundary clumping estimated by Morisita's overlap index (which estimates the degree of boundary overlap between a pair of species: Leibold & Mikkelson, 2002). These three aspects of the matrix (coherence, turnover, and limits) would characterize each co‐occurrence pattern (nested subsets, checkers, Clementsian gradients, Gleasonian gradients, uniformly spaced gradients, and randomness) predicted via EMS (Leibold & Mikkelson, 2002). More recently (in 2010 by Presley et al., 2010), the idea of “boundary clump” has been introduced in the nested pattern process (which was originally developed only for turnover elements; Figure 1). Another point that was raised in the same paper is that of quasi‐structure (Figure 1), characterized by a less evident pattern and communities with a weak species–environmental gradient relationship (Presley et al., 2010). Accordingly, the nested subset is now partitioned by more three patterns (Hiperdispersed, Random and Clumped specie loss), raising the number of co‐occurrence patterns to eight.

Analysis of species co‐occurrence patterns using EMS elements is effective because it assesses associations between communities and local or regional conditions and elucidates which mechanism underlies the metacommunity structure. Thus, EMS can be used to infer the formative historical and biogeographical processes of metacommunities (Brasil et al., 2017; Henriques‐Silva et al., 2013; Leibold et al., 2004; Leibold & Mikkelson, 2002; Presley et al., 2010; Presley & Willig, 2010). In consequence, checkerboard patterning is expected in communities with higher intraspecific competition and low levels of relationship to environmental variables. In contrast, turnover is frequently observed in communities where the environmental gradient has strong effect on the species pool; it can be classified into three species loss patterns (Leibold et al., 2004): (a) species loss occurring in discrete blocks and contiguous boundaries of species’ distribution classified as Clementsian metacommunities (Leibold et al., 2004; Figure 1). It is observed when the metacommunity is formed by discrete geographic blocks of communities. These blocks are formed by sets of species that facilitate distribution pattern distinction; (b) species loss along an environmental gradient (Figure 1), where little or no overlap tolerance exists between species distributed along the gradient (Leibold et al., 2004); (c) species loss in a nonpredictable manner, indicating that the structuring environmental gradients is not necessarily the same for each species (Gleasonian pattern, Leibold et al., 2004; Presley et al., 2010; Figure 1).

An EMS approach must consider both satellite species (Pandit et al., 2009) and trophic guilds. Species can be classified as core or satellite (Hanski & Gyllenberg, 1993). Core species will be more abundant and widely distributed along the studied environmental gradient, that is, present in a greater number of the sites occupied by the metacommunity, and may be responsible for the observed distributional pattern, which is generally nonrandom (Hanski & Gyllenberg, 1993; Ulrich & Zalewski, 2006). In contrast, satellite species will be sparsely distributed in the metacommunity, occur infrequently, and, usually, display a random distribution pattern (Livingston & Philpott, 2010; Storch & Šizling, 2002; Ulrich & Zalewski, 2006). They may be important to nested patterns of co‐occurrence, but there is no consensus on this. If the metacommunity were to be a set of species structured by a variety of mechanisms, the EMS procedures would fail to find a pattern of co‐occurrence (Leibold & Mikkelson, 2002). In such cases, the separate analysis of each trophic guild may be appropriate.

Given the variety of patterns of species co‐occurrence, how they might be interpreted, and what could thus be inferred concerning the mechanisms underlying metacommunity structure, the goals of the current study were to identify: (a) patterns of stream fish species co‐occurrence in the Paraná River basin, (b) relationships between species co‐occurrence patterns and spatial (geographic distribution of sampled communities) and environmental gradients, and (c) consistency of metacommunity species co‐occurrence patterns, considering core species and trophic guilds from the species pool. The hypothesis tested is that the metacommunity formed for the species pool and core species will display a nested pattern, structured by the ME mechanism. This is expected because some stream fish species have both low tolerance to environmental gradients and low dispersion ability. Such characteristics tend to create species subsets (once the populations are clustered and speciation may occur) in a large and old drainage, like the Paraná River basin (201.3 MYA: Iriondo et al., 2007). In contrast, those fish species with greater dispersion ability are more likely to colonize new sites. Such new sites may, or may not, have the environmental characteristics favorable to the species, and so such new populations may become established or go locally extinct. This process (colonization‐local extinction) is dynamic and occurs all the time (Leibold et al., 2004). Such characteristics (species presence related to environmental gradient and low dispersion abilities) lead to species with greater dispersal capacities displaying a nested pattern, and being influenced by the ME mechanism, which represents a tradeoff between species with specific environmental requirements and those with high dispersal capacities. If the PD mechanism is operative, trophic guilds will show a checkerboard pattern, with competition as a filter.

## 2 MATERIALS AND METHODS

### 2.1 Delimiting study metacommunity boundaries

The use of a valid closed system is essential for effective metacommunity analysis, be it ecological or biogeographical (Leibold et al., 2004; Leibold & Mikkelson, 2002; Presley et al., 2010). Accordingly, it was important to accurately delimit the system under study. Metacommunity delimitation depends on the dispersal ability of the species in the pool. In practice, a set of communities (a metacommunity) is delimited assuming that all species present may (potentially) colonize all communities (sites).

Many methods have been suggested to study this (Carstensen et al., 2013; Lepš, 2001; Naeslund & Norberg, 2006; Pärtel et al., 1996). The fish fauna of the Upper and Lower Paraná section forms a distinct biogeographic unit, both sections sharing many of the 250 species present in the basin and displaying a great variation of the environmental conditions (Abell et al., 2008). These characteristics allow the fish fauna of this basin to be considered as an ichthyological metacommunity.

**Figure 2:**
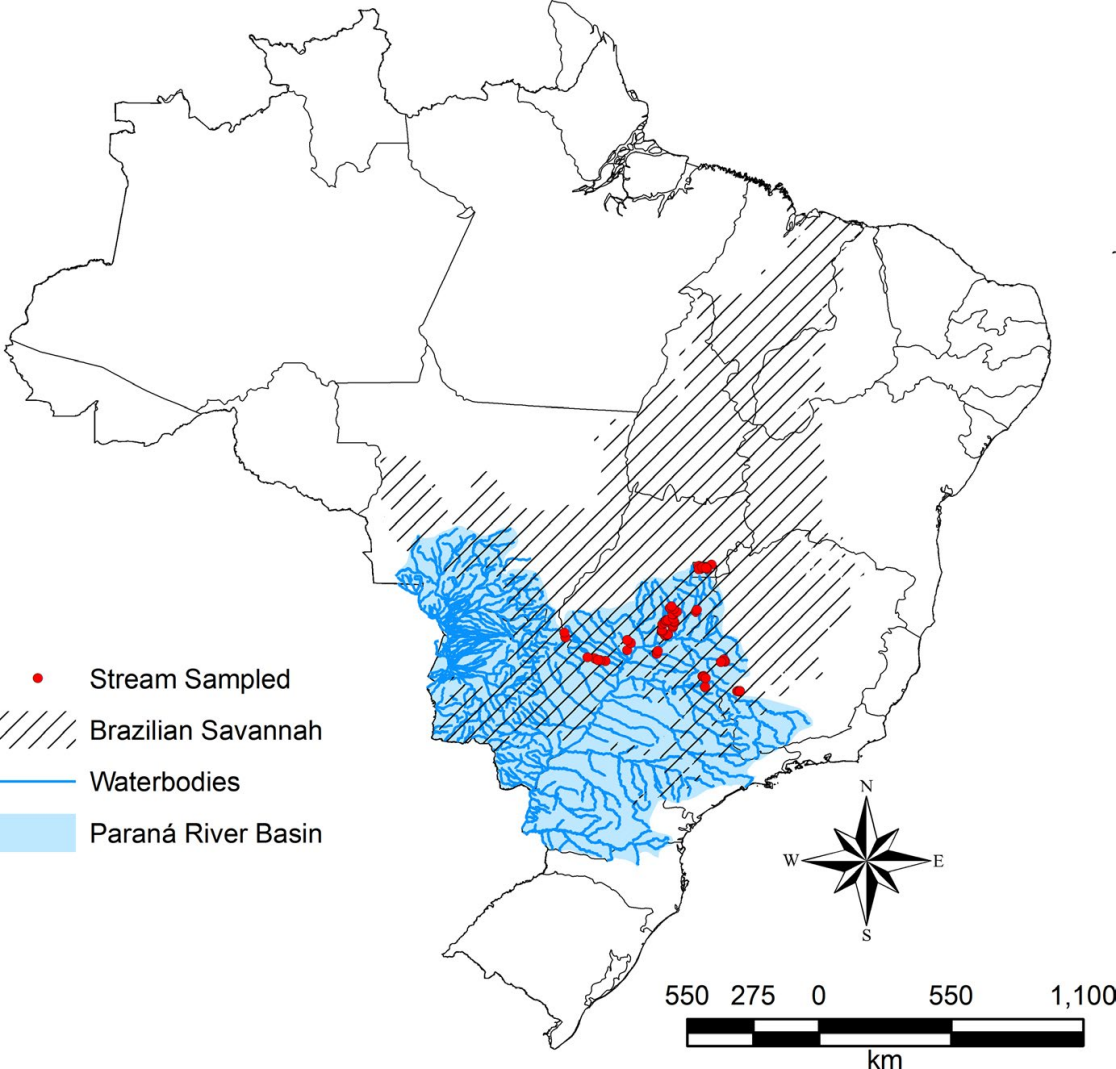
Fish metacommunity used in the EMS analysis. Red points indicate the spatial distribution of streams sampled (communities) in the Paraná River basin, Brazil. The shaded area represents the vegetation cover (Brazilian savannah)

In the current study, the metacommunity is bounded by the dendritic fluvial systems and network connectivity of 1st to 3rd order streams of the Paraná River basin (Figure 2). Accordingly, each sampled stream was considered as a community and the set of streams as the metacommunity. This approach is justified by the low ichthyofaunal similarity observed between streams (1st–3rd order) and rivers (>4th order; Araújo & Tejerina‐Garro, 2007; Melo et al., 2009; Dias & Tejerina‐Garro, 2010; Vieira & Tejerina‐Garro, 2014).

### 2.2 Database

A fish fauna database from 66 sampled streams within the Paraná River basin was used (Figure 2). A sampled stream was included in the database if it met the following requirements: (a) fish collection was carried out in a 1st–3rd order stream; (b) the sampled stream stretch was >50 m and georeferenced; (c) the fish sampling method used was electric fishing, trawl nets, and/or hand nets; (d) sampling comprised of a single sample site per stream; (e) a stream‐specific species list was available; (f) environmental variables (turbidity, conductivity, pH, dissolved oxygen, water velocity, stream channel depth and width) were measured in situ at three or more points in each stream; (g) fish sampling, transport, and preservation of sampled specimens were authorized by the *Sistema de Autorização e Informação em Biodiversidade* (SISBIO), Instituto Chico Mendes de Conservação da Biodiversidade, Ministerio do Meio Ambiente; and (h) streams were located in the same type of vegetation cover (the Brazilian savannah called Cerrado Biome, in this case). Constraining the data for streams under the same vegetation condition ensured that the features linked to vegetation type, such as substrate and temperature, are unlikely to differ greatly, so decreasing the amount of variability (Vieira & Tejerina‐Garro, 2020). The fish fauna dataset is available at: https://doi.org/10.5061/dryad.m63xsj3z7.

Using the specialized literature, component species were classified by trophic guild (detritivores, insectivores, and omnivores). Next, a species pool for each trophic guild was constructed and analyses performed to identify patterns; only pools with more than 15 species were considered (Table S2). To classify each species as core or satellite, the occurrence percentage of each species and the mean species occurrence in the metacommunity was calculated; species with occurrence greater than the mean were classified as core, less that this as satellite (Table S2).

### 2.3 Statistical analysis

**Figure 3:**
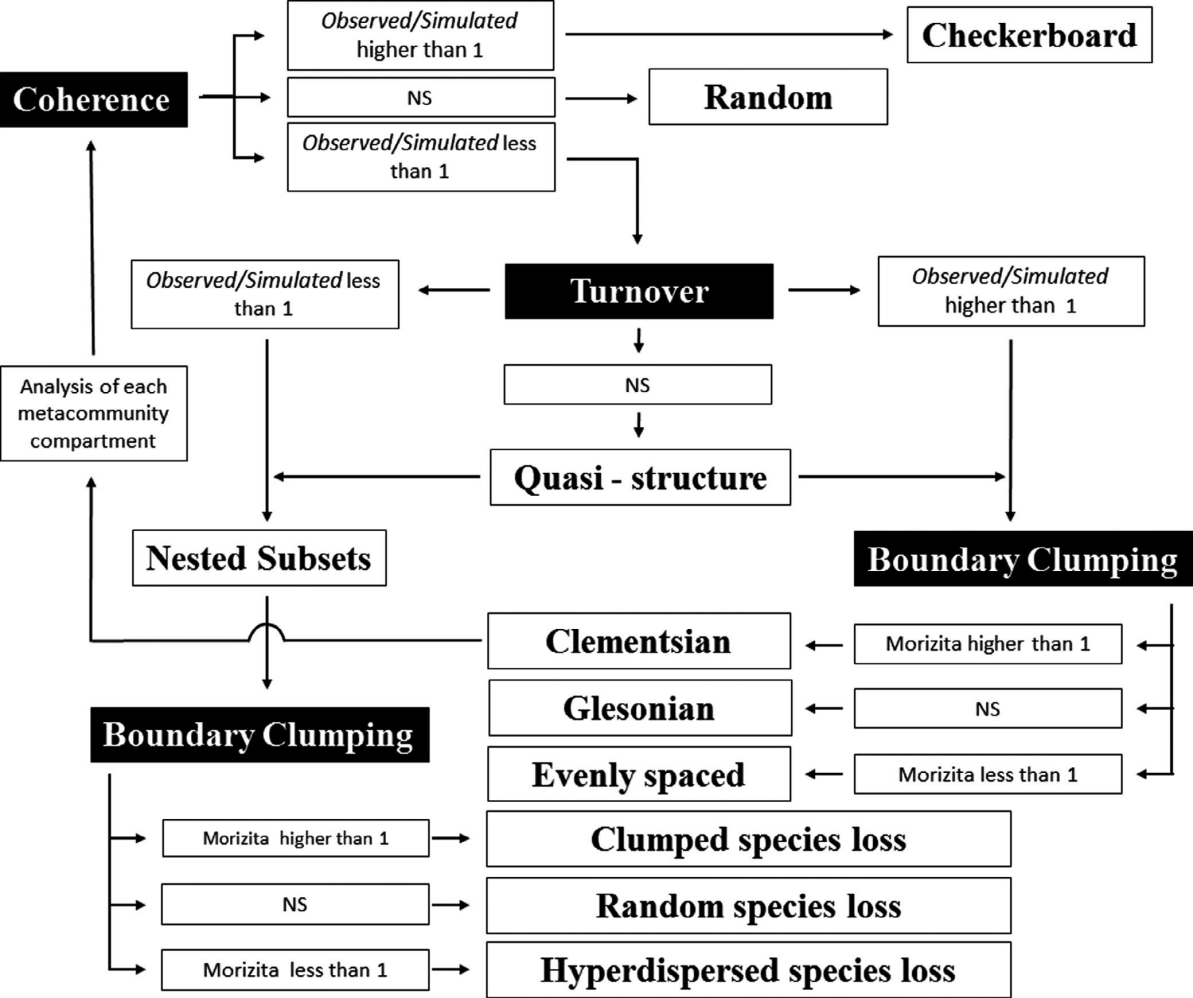
Flowchart showing all possible results from the co‐occurrence pattern analysis. Adapted from Leibold and Mikkelson (2002) and Presley et al. (2010)

Determination of other species co‐occurrence patterns was based on the analytical procedure described by Presley et al. (2010; Figure 3). For this, data were organized in a doubly ordered incidence matrix, with species richness (sum of rows) and occurrence (sum of columns) in the communities (Leibold & Mikkelson, 2002). This procedure grouped species by similar occurrence and communities by similar richness creating a gradient within the matrix (Presley & Willig, 2010). Double matrix ordering is the best procedure to identify species co‐occurrence patterns in the absence of an explicit gradient (Leibold & Mikkelson, 2002). Incidence matrix coherence was measured by embedded absences in the ordinated matrix (Leibold & Mikkelson, 2002). Turnover was estimated by substitution number in pairs of species, with a higher value indicating a turnover in the analyzed metacommunity (Leibold & Mikkelson, 2002). Boundary clumping was estimated with Morisita's overlap index, which calculates the species distribution overlaps (Leibold & Mikkelson, 2002; Figure 3).

The calculated EMS (coherence, turnover, and boundary clumping) encompassed all species co‐occurrences patterns. Pattern identification allocation considered the following: (a) defined matrix coherence; if the coherence was statistically significant and its value was higher than that of a randomized incidence matrix, the pattern was determined as checkerboard, if the coherence value was lower it was considered a turnover element, while if the coherence was not significant, the pattern was identified as random (Leibold & Mikkelson, 2002; Figure 3); (b) for the turnover element, two major patterns (nested subsets and boundary clumping) were analyzed (Figure 3). If turnover was statistically significant, and the observed value was lower than the randomized matrix, the system was identified as a nested subset with three possible patterns (hyperdispersed, random, and clumped species loss; Figure 3); if the coherence value was greater than random, it was considered as a boundary clumping element with three possible patterns (Clementsian, Gleasonian, and evenly spaced; Figure 3). If the turnover was not statistically significant, it was identified as a quasi‐structure and the analysis proceeded to the next step (Presley et al., 2010; Figure 3); (c) Morisita's overlap index was calculated irrespective of whether the data were defined having nested subsets or boundary clumping (Leibold & Mikkelson, 2002; Figure 3). The resulting index values were interpreted as follows: an index value > 1 and statistically significant (*p*‐value <.05) indicated a Clementsian pattern in boundary clumping, and clumped species loss in nested subsets; an index value <1 and statistically significant (*p*‐value <.05) was considered as an evenly spaced pattern in the boundary clumping, and as hyper dispersed species loss in a nested subsets; a nonsignificant Morisita's index (*p*‐value >.05) indicated a Gleasonian pattern in boundary clumping, and random species loss in nested subsets (Leibold & Mikkelson, 2002; Presley et al., 2010; Figure 3). After species co‐occurrence patterns identification, the matrix was restructured to show pattern presence as colored cells and absence as noncolored ones. The statistical significance of all elements was tested with a null model using the lines and columns with fixed sum and equal to richness, and original metacommunity abundance distributions (Presley et al., 2010).

To identify the environmental gradients underling the species co‐occurrence patterns in the metacommunity, a multiple regression tree (MRT) was constructed. For this, the variable response the first axes scores resulting of a correspondence analysis (CA) was used, using the species data matrix (presence and absence). The CA method is used for double matrix ordering in EMS analysis (Presley et al., 2010). Values for water turbidity, conductivity, pH, dissolved oxygen, and temperature and those of the stream channel width and depth were used as predictors for the MRT. All analysis was performed in R software (R Development Core Team, 2017), using the function metacommunity for EMS, package *metacom* (Dallas, 2014) and function *rpart* for MRT, package *rpart* (Therneau & Atkinson, 2019). All procedures were performed on the metacommunity, a category that included all species in the pool, core species, and trophic guilds (Figure S1).

## 3 RESULTS

The pool for analysis comprised of 143 species distributed across 66 streams. Fish species richness per stream varied from 1 to 27 species (Table S1). Four metacommunities were determined based on the total pool of species and the detritivores, insectivores, and omnivores pool. These had 66, 52, 66, and 56 discrete communities (streams) with 143, 40, 63, and 27 species, respectively. All metacommunities had statistically significant higher coherence values than those of null models (Table 1). The species co‐occurrence pattern was quasi‐structured with nested subsets and clumped species loss (Table 1), because the turnover was not statistically significant (its value was lower than the null model), and Morisita's overlap index >1 (Table 1). In contrast, the core species metacommunity had 69 communities and 94 species displaying statistically significant coherence and values higher than the null model (Table 1). Because the turnover was not statistically significant (its value was higher than that of the null model), and Morisita's overlap index >1, the pattern was quasi‐structured and Clementsian (Table 1).

**Table 1:** Statistics of the elements of metacommunity structure (EMS) for the fish metacommunity from the Paraná River Basin, Brazil. *p* < .05; *df* = degree of freedom

Element	Metacommunity				
	Pool	Detritivore	Insectivore	Omnivore	Core
Species richness	143	40	63	27	94
Coherence					
Observed	3,188	430	937	259	2,143
Randomized	5,936	697	1,774	371	3,814
Observed/Randomized	0.537	0.616	0.528	0.698	0.561
*p*	<.001	<.001	<.001	.039	<.001
Turnover					
Observed	874,297	20,448	73,385	9,296	462,675
Randomized	1,089,300	36,009	138,166	17,085	374,948
Observed/Randomized	0.802	0.567	0.531	0.544	1.233
*p*	.576	.09	.112	.066	.47
Boundary clumping					
Morisita's index	7.565	3.42	3.44	3.08	5.05
*p*	<.001	<.001	<.001	<.001	<.001
*df*	140	37	60	24	91
SEM	Clumped species loss	Clumped species loss	Clumped species loss	Clumped species loss	Clementsian

**Figure 4:**
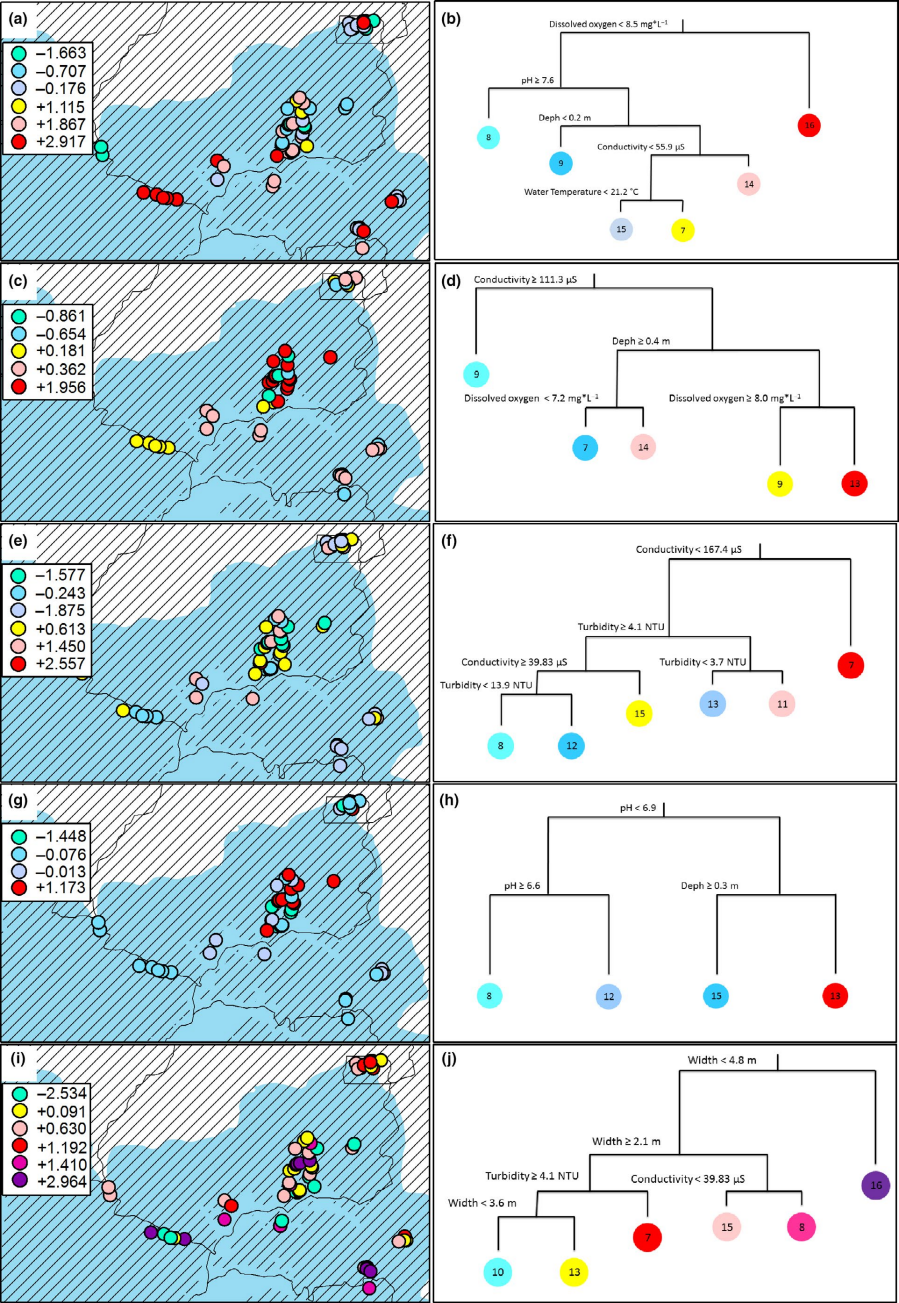
Sampling point spatial distributions (left) classified according to the multiple regression tree (right). Pool (a, b), detritivores (c, d), insectivores (e, f), omnivores (g, h), and core (i, j) metacommunity. Legend values represent mean group scores for the correspondence analysis (CA) first axis. Red = fish communities related to water dissolved oxygen; light blue = water pH; blue = stream channel depth; pink = water conductivity; dark yellow = water temperature

The observed pool species co‐occurrence pattern was related to five environmental conditions. Fish communities from 16 streams were associated with higher water dissolved oxygen values (>8.5 mg/L), while those of five streams were related to low values (<8.5 mg/L). One set of the fish community was observed in eight streams with pH > 7.6, a second one in nine streams with channel depth > 20 cm, a third one in 14 streams with conductivity values >55.9 µs/cm, a fourth one in 15 streams displaying water temperature values < 21.2°C, and the final community from seven streams with water temperature >21.2°C (Figure 4a,b).

For the detritivore metacommunity (Figure 4c,d), nine stream fish communities were associated with conductivity values >111.3 µs/cm and four with low ones <111.3 µs/cm. The fish community was associated with 21 shallow streams (depth < 40 cm), one set of which (14 streams) also showing an association with water dissolved oxygen values >7.3 mg/L, while another part (seven streams) were linked to low dissolved oxygen values <7.3 mg/L. Another part of the fish community showed an association with deep streams (>40 cm), displaying higher dissolved oxygen values >8.0 mg/L (nine streams) or low dissolved oxygen values <8.0 mg/L (13 streams; Figure 4c,d).

Insectivores showed six sets of fish communities related to two environmental variables (Figure 4e,f). One set, composed of nine streams, was related to high water conductivity values (>167.4 µs/cm), with another set composed by five subsets related to low value (<167.4 µs/cm). These latter were associated with high water turbidity values (>3.7 NTU; 11 streams), to turbidity values between 4.1 and 3.7 NTU (13 streams) and to turbidity values >4.1 NTU (24 streams). One set of fish communities (15 streams) was associated with high water conductivity values (>39.83 µs/cm) and two sets with low values (<39.83 µs/cm). Of the latter, one was associated with high turbidity values (>13.9 NTU; 12 streams) and the other with low values (<13.9 NTU; eight streams; Figure 4e,f).

Omnivores had four sets of fish communities (Figure 4g,h). One set related to deep channel streams (>30 cm; 13 streams), and another to shallow streams (<30 cm; 15 streams); both sets were associated with neutral or alkaline water (pH > 6.9). The third and fourth sets were composed of fish communities associated with acid water streams, that is, pH < 6.6 (12 streams), and pH values varying from 6.6 to 6.9 (eight streams), respectively (Figure 4g,h).

The core species metacommunity showed seven sets of fish communities. These showed relations to three environmental conditions (Figure 4i,j). One set was related to wide stream channels (>0.48 m; 16 streams), while two sets where associated with stream channel widths between 210 and 480 cm (23 streams). Of the letter, one set was also associated with high water conductivity values (>39.8 µs/cm; eight streams) and the other with low ones (<39.8 µs/cm; 15 streams). Three sets were associated with narrow stream channels (2.1 to 5.6 m), of which one set was also linked with stream channels with high water turbidity values (>4.1 NTU; seven streams) and the other two sets with low turbidity values (<4.1 NTU). Of the latter, one set was associated with narrow stream channels (<3.60 m; 10 streams) and the other with wide streams channels (>3.60 m; 13 streams; Figure 4i,j).

## 4 DISCUSSION

The results showed that environmental and spatial gradients are important for the species distribution as well as for its dynamic. This becomes more evident when communities are deconstructed into groups with greater inter‐specific ecological relationships, for example, to obtain and consume food resources or in within‐landscape dispersion abilities. The metacommunity (the species pool) present in Paraná River Basin streams shows significant coherence, suggesting the presence of an underlying environmental/spatial gradient (Livingston & Philpott, 2010; Mouquet & Loreau, 2002; Presley & Willig, 2010). When the metacommunity is deconstructed into trophic guilds and core species, the coherence (absences embedded in presences) remains consistent across all metacommunities, suggesting that environmental gradients structure the sampled stream fish metacommunities of the basin. In environments with more or less discreet community boundaries, Clementsian structures are predominant and have better fit, which indicates the importance of species turnover between locations (Erős et al., 2014). For turnover, nested pattern was identified in all metacommunities, except the core species metacommunity. This suggests that, alongside the environmental gradient underlying the metacommunities, there are species in the pool with great dispersal ability (Presley & Willig, 2010), represented by the satellite species. Their presence is supported by the results of the core species EMS. Core species had a Clementsian pattern, that is, one characterized by an environmental gradient and the absence of strongly dispersing fish species in the pool (Leibold et al., 2004).

The discordant results between the core and the other metacommunities can be explained by the coexistence of two species groups: a group with an old colonization history (core species) and another with a more recent history of colonization (satellite species); the core group is composed by species with similar physiologies and evolutionary histories (Henriques‐Silva et al., 2013; Livingston & Philpott, 2010; Mouquet & Loreau, 2002; Presley & Willig, 2010). The core species (the oldest species in the metacommunity) display a stable process of dispersion, colonization, and establishment within the communities and, consequently, the species–environmental gradient relationship is strong, so favoring the Clementsian pattern of species co‐occurrence. On the other hand, the satellite species (the more recent species in the metacommunity) are in an ongoing process of dispersion, colonization, and establishment, which results in higher local extinction and lower establishment rates than for the core species. Both processes mask the relationship with the environmental gradient and could form a random pattern that remains to be tested for. In fact, the nested subsets patterns of all metacommunities, except that of core species, can be explained by species dispersion mechanisms. Nested subsets are formed when a set of species is related to environmental gradients (the core species), but part of this either has a less obvious relationship with environmental gradients or displays strong dispersal abilities, "strong" meaning the capacity to mask the species turnover within a nested subset (satellite species). The impact of the latter species on species co‐occurrence patterns (Livingston & Philpott, 2010) has been previously described for both aquatic insects (Heino & Soininen, 2010) and plants (Tuomisto et al., 2003).

The species‐sorting (SS) and mass‐effect (ME) mechanisms proposed by Leibold et al. (2004) can explain the patterns of species co‐occurrence in stream fish communities. Both mechanisms link metacommunities composition to environmental conditions, differing in the role of species dispersal capacity (Leibold et al., 2004). In fact, it seems that the difference between SS and ME relates only to the dispersal ability of species in the pool. With the ME mechanism, species not only have poor dispersal capacity (dispersion occurs, but at low rates and cannot modify the overall pattern), but also high dispersal capacity; thus, they can modify the pattern. This is possible, because ME generates patterns related to an environmental and spatial gradient (like nested subsets), while SS generates only those related to environmental gradients (Leibold et al., 2004). High species dispersion ability creates a dynamic akin to a source‐sink situation, where species with high dispersal ability can colonize sites without suitable environmental conditions for the long‐term establishment of populations, increasing the local extinction rates at these sites, so masking the turnover in a nested subsets pattern (Mouquet & Loreau, 2002).

Metacommunities formed by all species in the pool and classified into trophic guilds were best explained by ME. This occurs because these communities had nested subsets, which were related to an environmental gradient and characterized by species with different dispersal abilities. However, when satellite species were removed, the EMS may appear to be structured by SS, that is, metacommunity composition is only related to the environmental gradient. Despite this, in the current study, the core metacommunity was explained by a SS mechanism and displayed a Clementsian pattern. This suggests that species dispersion in the metacommunity cannot be ignored, because the differing dynamics within its structure can impact species classification, vis‐à‐vis their core or satellite designation (Cottenie, 2005; Erős et al., 2017; Leibold et al., 2004), but not the species distribution pattern (Cottenie, 2005). The interaction between environment and spatial processes observed for the stream fish communities in this study (ME pattern) has also reported for lake (Magnuson et al., 1998), floodplain fish communities (Henriques‐Silva et al., 2013), and aquatic invertebrate communities (Göthe et al., 2013). ME has been considered the principal mechanisms for structuring natural metacommunities (Cottenie, 2005). In dendritic networks, like streams, ME is the most powerful mechanism because both connectivity and the potential of dispersion are high (Göthe et al., 2013), thus favoring the ME mechanism (Leibold et al., 2004). Soininen (2014, 2016) and Soininen et al. (2018) reported that the degree of SS varies according to the trophic group, having low values in decomposers and herbivores (beta diversity) fresh water, and high ones in trophic groups of marine estuaries.

In this paper, the studied streams were in a hydrographic basin covered throughout by a similar vegetation type (Brazilian savannah called Cerrado). Thus, the variation shown by the regression tree results can be related to the idiosyncratic characteristics of each sampling site, supporting the participation of the environmental gradient in the ME pattern. On the other hand, nested patterns and dispersion can be explained by the low connectivity between sampling sites, and the high turnover at sites with elevated environmental heterogeneity, that is, the differences between the rainy and dry season observed in the Brazilian savannah (Henriques‐Silva et al., 2013; Magnuson et al., 1998). The rainy season is accompanied by a strong increase in water flow, with the result that many fish may be carried downstream. In the dry season, water flow is much reduced, and fish may recolonize the upstream sites. However, within‐course variations in ecological conditions of streams and rivers connecting such sites act as a filter, decreasing the overall colonization dynamic (Benone et al., 2018). In general, environmental variables are more important for determining metacommunity structure than spatial ones, resulting in a major influence of the anthropogenic effects (Erős et al., 2012).

Nested subsets patterns within fish communities are associated with sites possessing high levels of temporal or geographic environmental heterogeneity (Fang & Stefan, 2000; Fernandes et al., 2013; Henriques‐Silva et al., 2013; Magnuson et al., 1998). Such nested subsets have been related to the temperature and humidity variation in terrestrial gastropods (Bloch et al., 2007), and to water volume and stream heterogeneity caused by glacial melting for fish in temperate lakes (Fang & Stefan, 2000; Magnuson et al., 1998). For tropical fishes, the nested subsets can be explained by the annual flood pulse, which influences hydric basin heterogeneity, which is a key factor in determining fish diversity patterns (Astorga et al., 2011; Vinson & Hawkins, 2003). A possible mechanism underlying flood pulse‐derived nested patterns is the loss of some species during the rainy season and subsequent recolonization in the dry season.

During the rainy season, increases in water volume and flow rates tend to scour streams, decreasing the heterogeneity and diversity (Benone et al., 2018). In the dry season, the water volume return to a “normal” condition, and deposition of sediment, leaves, and twigs, among others, increases local heterogeneity, so favoring site recolonization by fish (Benone et al., 2018). However, under circumstances of prolonged drought, the diversity and abundance of fish species can be drastically reduced (Driver & Hoeinghaus, 2016). The colonization process is performed more efficiently by abundant species, according to the neutral approach, or by species best adapted to particular environmental conditions, according to the niche approach. Consequently, less abundant or poorly adapted species would be less efficient than other species at forming a nested pattern. This mechanism is consistent with the idea of the coexistence of two sets of species, the core species adapted to the environmental condition and displaying Clementsian patterns of co‐occurrence, and the satellite species, less adapted to the environmental conditions and with a distribution pattern more closely reflecting spatial patterning, and the neutral dynamic (not yet tested). The conjunction of these two sets results in a nested subset pattern.

It is concluded that core species with Clementsian patterns of co‐occurrence are structured by SS mechanisms. Each pattern is determined by the environmental conditions and fish species with a low rate of dispersion. In contrast, satellite species tend to have strong dispersal abilities and may be structured by the neutral process (patch dynamic or neutral). Because of this, when the whole metacommunity (core + satellite species) is analyzed, a nested subset pattern is observed, with ME operating as the structuring mechanism and distribution of fish community species correlated with local (core species) and regional (satellite species) conditions. The loss of Clementsian (core species) and clumped species (all the other metacommunities) indicate the presence of barriers responsible for the formation of subsets of communities within the metacommunity (Alves‐Martins et al., 2019). In the case of Paraná River streams, physicochemical water characteristics and main channel morphology was identified as barrier for fish species as indicated by MRT.

## CONFLICTS OF INTEREST

There are no conflicts of interest.

## AUTHOR CONTRIBUTION

**Thiago Bernardi Bernardi Vieira:** Formal analysis (lead); Writing‐original draft (lead). **Leandro Schlemmer Brasil:** Writing‐review & editing (equal). **Liriann Chrisley Nascimento Da Silva:** Writing‐review & editing (equal). **Francisco Leonardo Tejerina Garro:** Writing‐review & editing (equal). **Pedro De Podestá Uchôa de Aquino:** Writing‐review & editing (equal). **Paulo Santos Santos Pompeu:** Writing‐review & editing (equal). **Paulo De Marco:** Supervision (lead).

## Supporting information


**Table S1**

**Table S2**

**Fig S1**

## Data Availability

Data are available as supplementary material in Vieira, Thiago Bernardi et al. (2020), Elements of fish metacommunity structure in Neotropical freshwater streams, Dryad, Dataset, https://doi.org/10.5061/dryad.m63xsj3z7.
