# Supplementary figures and images for: Elements of fish metacommunity structure in Neotropical freshwater streams
Source: Ecol Evol. 2020 Oct 15;10(21):12024–35. doi: 10.1002/ece3.6804 (PMC7663076; doi:10.1002/ece3.6804)

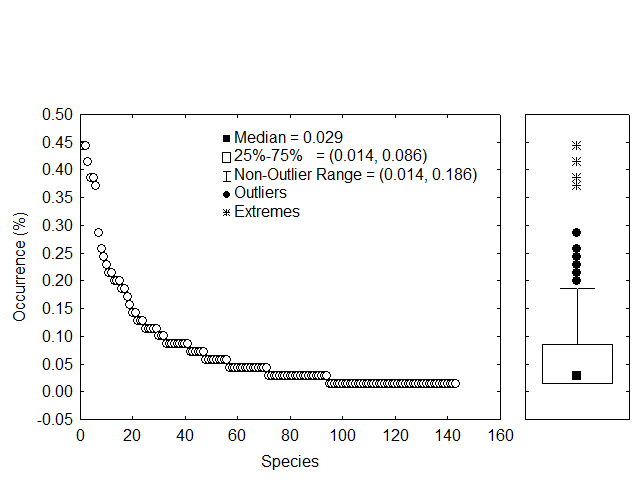

Supplement: Supplementary file 3 — Fig S1 [file ECE3-10-12024-s003.tif]
